# Robotic Versus Open Pancreaticoduodenectomy: A Single-Center Analysis of Safety and Efficacy Using Inverse Probability of Treatment Weighting

**DOI:** 10.3390/cancers17121916

**Published:** 2025-06-09

**Authors:** Mariano Cesare Giglio, Silvia Campanile, Gianluca Rompianesi, Giuseppe Loiaco, Riccardo Aurelio Nasto, Roberto Montalti, Roberto Ivan Troisi

**Affiliations:** Division of Minimally Invasive and Robotic HPB Surgery and Transplantation Service, Federico II University Hospital, 80131 Naples, Italy; mariano.giglio@unina.it (M.C.G.); silvia.campanile@unina.it (S.C.); gianluca.rompianesi@unina.it (G.R.); giuseppe.loiaco@unina.it (G.L.); riccardoaurelio.nasto@aoufedericoii.unina.it (R.A.N.); roberto.montalti@unina.it (R.M.)

**Keywords:** pancreaticoduodenectomy, robotic surgery, open surgery, inverse probability of treatment weighting

## Abstract

**Background/Objectives:** Robotic surgery has emerged as a promising alternative to traditional approaches, offering enhanced precision and ergonomics. However, its application to pancreaticoduodenectomy (PD) remains controversial due to technical complexity and a steep learning curve. This study aims to evaluate the perioperative outcomes of robotic versus open PD at a single high-volume center, using inverse probability of treatment weighting (IPTW) to control for confounding variables. **Methods**: A retrospective, single-center analysis was conducted on patients who underwent PD between January 2020 and December 2024. Perioperative and oncological outcomes were compared using IPTW, aiming to balance potential confounding variables. The primary endpoint was 90-day postoperative mortality; secondary endpoints included operative time, estimated blood loss, major postoperative complications (Clavien-Dindo grade ≥ IIIa), and length of hospital stay. **Results**: Seventy-four patients were included in the intention-to-treat analysis (open: *n* = 31; robotic: *n* = 43). After IPTW adjustment, baseline characteristics were balanced across the groups. Operative time was significantly longer in the robotic group (median 540 vs. 479 min, *p* = 0.009). No significant differences were observed in 90-day mortality (4.9% both groups, *p* = 0.998), postoperative complications ≥ IIIa, or histopathological outcomes, including R0 resection and lymph node yield. Trends toward reduced estimated blood loss and shorter hospital stay in the robotic group did not reach statistical significance. **Conclusions**: Robotic pancreaticoduodenectomy demonstrates comparable safety and oncological efficacy to the open approach, with longer operative times but potential perioperative advantages. These findings support the continued integration of robotic techniques even in medium-volume centers with appropriate expertise.

## Simple Summary

This study aimed to compare the outcomes of robotic and open pancreaticoduodenectomy in a medium-volume center, using inverse probability of treatment weighting to reduce the risk of selection bias. Robotic surgery was found to have a longer operative time but achieved similar safety and efficacy outcomes to the open approach. Although trends suggested possible benefits such as less blood loss and shorter hospital stay with robotic surgery, these differences were not statistically significant. Our findings support the safe and effective adoption of robotic pancreaticoduodenectomy in a medium-volume center.

## 1. Introduction

Open surgery represents the standard approach for pancreaticoduodenectomy (PD) in many institutions, given the technical complexity of this procedure. The introduction of minimally invasive techniques has progressively expanded the surgical options also in pancreatic surgery [1,2]. Following the early suspension of the LEOPARD-2 trial investigating the safety of laparoscopic PD [3], the robotic approach has gained increasing interest due to its technical advantages, including enhanced visualization, improved ergonomics, and greater precision through articulated instruments and tremor filtration [4].

Current literature suggests that robotic PD can achieve oncological and functional outcomes comparable to, or in selected cases better than, conventional approaches [5,6,7]. Nevertheless, robotic PD remains technically demanding. Limited working space, the need for meticulous dissection near major vessels, and the fragile nature of pancreatic tissue contribute to the complexity of these procedures. Also, the reconstructive phase is particularly challenging, and postoperative pancreatic fistula (POPF) remains the most frequent and feared complication [8].

Importantly, outcomes in PD are closely related to institutional case volume and surgeon experience, with high-volume centers demonstrating lower morbidity and mortality rates [9,10]. These findings have led to increasing efforts toward centralization of PD, particularly for minimally invasive and robotic approaches. The adoption of robotic PD has been slower than in other surgical domains, mainly due to the steep learning curve, increased operative times, and the concentration of expertise in high-volume centers [11,12,13]. Efforts to standardize indications and techniques are ongoing, supported by recent evidence-based guidelines [14].

On this background, the present study aims to compare the perioperative outcomes of robotic-assisted versus open pancreaticoduodenectomy at a single medium-volume center during the implementation phase of a robotic pancreatic surgery program using inverse probability of treatment weighting analysis.

## 2. Materials and Methods

### 2.1. Study Design and Study Population

This was a single-center, observational, retrospective study comparing outcomes between patients undergoing open versus robotic pancreaticoduodenectomy using inverse probability of treatment weighting analysis.

All patients who underwent pancreaticoduodenectomy at our institution between January 2020 and December 2024 were identified from a prospectively maintained database. The inclusion criteria were the following: adult patients (>18 years); indication for pancreaticoduodenectomy for any disease. Exclusion criteria were patients with adjacent organ invasion or distant metastases. Patients were selected and analyzed according to an intention-to-treat principle, with patients selected for robotic pancreaticoduodenectomy and intraoperatively converted to open surgery analyzed within the robotic group.

An inclusive selection policy was adopted for the robotic-assisted approach to pancreaticoduodenectomy (PD). Since its introduction, all patients without absolute contraindications were considered eligible for robotic PD. Contraindications included the anticipated need for vascular resection, body mass index (BMI) ≥ 30 kg/m^2^, previous major supramesocolic surgery, and contraindications to pneumoperitoneum.

### 2.2. Data Collection and Definitions

The following parameters were analyzed retrospectively: operative time, estimated blood loss, blood transfusions, conversion to open surgery, postoperative complications (including postoperative POPF, bile leakage, delayed gastric emptying (DGE), post-pancreatectomy haemorrhage (PPH), length of postoperative hospital stay (LOS), and tumour pathology. Reoperation rates, 90-day readmissions, and 90-day mortality were also recorded. Postoperative complications were graded according to the Clavien-Dindo classification, with major complications defined as grade ≥ IIIa. POPF, DGE, and PPH were defined and graded according to the criteria of the International Study Group of Pancreatic Surgery [15,16,17]. R0 resection was defined as no malignant cells within 1 mm of the pancreatic stump and posterior resection margin. Our center was classified as a medium-volume center, considering the thresholds (10–30 resections/year) proposed by Balzano et al. [18]

### 2.3. Surgical Technique

#### 2.3.1. Open Pancreaticoduodenectomy

Open pancreaticoduodenectomy was performed through a bilateral subcostal laparotomy. After exploration and exclusion of distant metastases, a standard resection without pylorus preservation was carried out. The gastroduodenal artery stump was ligated or stapled one cm from the origin, wrapped with the fat coming from the round ligament. Reconstruction was achieved using a single jejunal loop brought into the supramesocolic compartment via a retromesenteric route, following a Child-type configuration. Pancreatojejunostomy, hepaticojejunostomy, and gastrojejunostomy were sequentially constructed. The modified Blumgart technique was routinely used for the pancreatic anastomosis [19]. The hepaticojejunostomy was created approximately 40 cm distal to the pancreatojejunostomy in a side-to-end fashion, using either interrupted or running sutures with polydioxanone (PDS), depending on the size of the bile duct. Gastrojejunostomy was performed either with a linear stapler or by hand-sewn double-layer technique using absorbable monofilament sutures, based on surgeon preference. Lymphadenectomy was performed according to standardized institutional protocols. Typically, two Blake-type drains were positioned near the pancreatic anastomosis, one Penrose drain below the pancreatic anastomosis; all drains were managed based on drain output and amylase levels. Open PDs were performed by two surgeons who had already completed the established learning curve for open pancreaticoduodenectomy.

#### 2.3.2. Robotic Pancreaticoduodenectomy

Robotic pancreaticoduodenectomy was performed using the da Vinci Surgical System, with the patient in the supine position with a 30-degree reverse Trendelenburg tilt. The phases of dissection of the uncinate process and retroportal lamina as well as pancreatic anastomoses were performed by the senior author (RIT), with an experience of 15 robotic PD before promoting this approach at this institution. This experience was acquired without participation in a formal training program, proctoring system, or mentorship at a high-volume center for robotic PD. After a staging laparoscopy aiming to exclude intrabdominal extra pancreatic disease, the resection and reconstruction phases were completed using robotic instrumentation. The surgical steps mirrored those of the open approach. Vascular dissection and division were performed using energy devices such as harmonic shears or Hem-o-Lok clips. All anastomoses were performed intracorporeally. Both pancreatojejunostomy and hepaticojejunostomy were carried out using techniques comparable to those employed in the open procedure. Gastrojejunostomy was constructed with a stapled technique. Drain placement and postoperative management followed the same principles as the open approach.

### 2.4. Statistical Analysis

The primary outcome was in-hospital mortality within 90 days postoperatively. Secondary outcomes included length of hospital stay, operative time, estimated blood loss, pathological resection margin status (R), number of lymph nodes retrieved, incidence of DGE, clinically significant POPF, bile leakage, postoperative bleeding requiring reintervention, Clavien-Dindo grade ≥ IIIa complications, 90-day reoperation, and readmission rates.

Continuous variables were reported as medians with interquartile ranges and compared using either the Student’s *t*-test or the Wilcoxon rank-sum test, based on data distribution. Categorical variables were presented as counts and percentages and compared using Pearson’s chi-squared test or Fisher’s exact test, as appropriate.

To minimize the impacts of potential confounders and selection bias, an inverse probability treatment weight analysis was performed [20]. Propensity score, that is the probability for each patient to undergo open or robotic pancreaticoduodenectomy, was calculated for each patient using logistic regression based on the following covariates: age, gender, body mass index, ASA Score, Fistula Risk score, malignant tumour, pancreatic adenocarcinoma, and preoperative chemotherapy. Patients with missing data for these covariates were excluded from the analysis, under the assumption of data missing completely at random. This assumption was considered acceptable in this context given the low proportion of missing data and the absence of identifiable patterns [21]. For each patient, a stabilized inverse probability treatment weight was calculated [22] and two weighted pseudo-populations of patients undergoing open or robotic pancreaticoduodenectomy were obtained with balanced confounding variables. Assessment of covariate balance between open and robotic groups before and after applying the IPTW was performed by calculating the Standardized Mean Difference (SMD), with a SMD value ≤ 0.1 indicating a good balance between the groups after IPTW [23].

Comparisons in the weighted dataset were adjusted for IPTW. For continuous outcomes, weighted linear regression was used; for categorical variables, weighted chi-squared or Fisher’s exact tests were applied depending on expected cell counts. All *p* values ≤ 0.05 were considered as statistically significant. Statistical analyses were conducted using R version 4.0.3 (R Core Team (2020). R: A language and environment for statistical computing. R Foundation for Statistica Computing, Vienna, Austria. URL https://www.R-project.org/).

## 3. Results

### 3.1. Patient Population and Baseline Characteristics

Between January 2020 and December 2024, a total of 90 patients underwent a pancreaticoduodenectomy at our institution, with an average of 18 procedures per year. After excluding laparoscopic-assisted and hybrid procedures, 74 patients who underwent either open or robotic-assisted pancreaticoduodenectomy were included in the intention-to-treat analysis. Of these, 31 patients received open surgery, while 43 underwent robotic-assisted procedures.

The characteristics of the entire cohort and baseline characteristics of the two groups are shown in Table 1. Conversion to open surgery was required in 10 patients (23%) undergoing robotic-assisted surgery. The reasons for conversions included the need to perform vascular resection (*n* = 4), difficulty in proceeding by robotic-assisted surgery (*n* = 3), and high-risk pancreatic (*n* = 3). No urgent conversions were required for intraoperative incidents or uncontrolled bleeding.

Although not statistically significant, imbalances between the open and robotic-assisted groups were observed in several variables, including ASA score, Fistula Risk Score, and the presence of biliary drainage at the time of surgery (SMD > 0.2), suggesting potential confounding factors that may bias outcome comparisons. Furthermore, a moderate imbalance (SMD > 0.1) was noted for other baseline variables (Table 1).

### 3.2. Covariate Balance Post-IPTW

**Figure 1:**
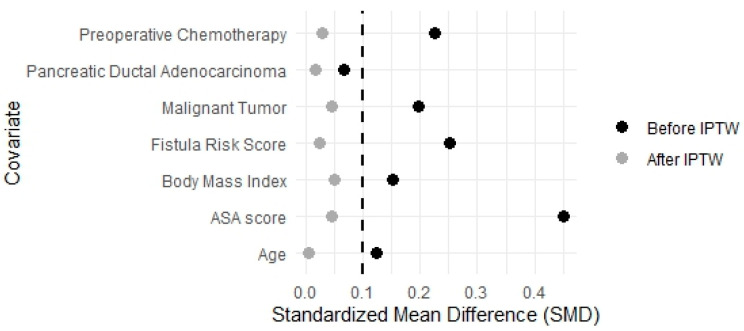
A love plot showing balance in covariates between the open and robotic-assisted groups before and after applying inverse probability of treatment weighting.

The application of IPTW generated two pseudo-populations of patients undergoing open (*n* = 31.27) and robotic-assisted (*n* = 42.75) pancreaticoduodenectomy (Table 2). In the weighted cohort, baseline covariate was substantially balanced across groups, with all SMDs below the 0.1 threshold (Table 2, Figure 1).

### 3.3. Perioperative Outcomes

Perioperative outcomes before and after IPTW are presented in Table 3. In the weighted cohort, operative time was significantly longer in the robotic-assisted group (540.00 min [470.71 to 600.00]) compared to the open group (479.40 min [393.18 to 498.00], *p* = 0.009). Although not statistically significant, there was a trend toward reduced estimated blood loss in the robotic-assisted group (*p* = 0.635).

Ninety-day mortality was identical in both groups (4.9%, *p* = 0.998). All mortality cases were related to POPF-related complications, including massive haemorrhage (*n* = 2) and sepsis (*n* = 1). No significant differences were observed in postoperative complications, including clinically relevant pancreatic fistula (*p* = 0.675), biliary leak (*p* = 0.334), and delayed gastric emptying (*p* = 0.464). Rates of postoperative bleeding requiring reoperation were also similar between the robotic (9.6%) and open (8.0%) groups (*p* = 0.797). Postoperative complications classified as Clavien-Dindo grade III or higher occurred at similar rates in the robotic (30.6%) and open (28.9%) groups (*p* = 0.880). The length of hospital stay tended to be shorter in the robotic group (17 vs. 20 days), although this difference was not statistically significant (*p* = 0.683). The 30-day readmission rate was also comparable between groups (*p* = 0.344), with a higher, though non-significant, trend observed in the open group (25.1% vs. 9.4%; *p* = 0.095). Histopathological analysis showed no differences between groups in the number of lymph nodes retrieved (robotic: 20 [13–27] vs. open: 20 [14–28]; *p* = 0.578) or in resection margin status (Table 3).

## 4. Discussion

This single-center, retrospective study compared the perioperative outcomes of robotic-assisted and open PD using IPTW analysis.

The findings from our analysis support the feasibility and safety of robotic-assisted pancreaticoduodenectomy in a medium-volume center, with perioperative outcomes largely comparable to those of the open approach. Although associated with a longer operative time, robotic PD demonstrated equivalent 90-day mortality, major morbidity, and clinically relevant postoperative pancreatic fistula to open surgery. In addition, a trend toward reduced estimated blood loss and shorter hospital stay was evident. These results are consistent with those reported so far in the literature [24,25,26,27,28].

The implementation of robotic PD remains associated with a substantial learning curve, particularly with regard to achieving proficiency. Recent multicenter studies have identified the inflection point for proficiency, that is the stabilization of major morbidity after approximately 62 to 93 cases [29,30]. In our study, all robotic cases were performed during the early phase of the institutional learning curve. Conversely, open procedures were carried out by surgeons who had already surpassed the learning curve for open PD. Therefore, the present results should be interpreted within this context.

It is important to emphasize that these results were achieved without a strict selection policy of patients for the robotic-assisted approach. Since the adoption of robotic PD at our institution, all patients without clear contraindications, such as anticipated need for vascular resection or large tumours, high BMI ≥ 30 kg/m^2^, prior major supramesocolic surgery, or contraindications to pneumoperitoneum, were considered eligible for the robotic approach. This inclusive strategy aimed to accelerate the learning curve of the procedure in a medium-volume pancreatic surgery center with established expertise in advanced minimally invasive liver procedures [29].

The availability of dedicated operating room slots for robotic hepatobiliary surgery, coupled with strong institutional and team commitment, facilitated the implementation of the robotic approach. A low threshold for conversion to open surgery was deliberately maintained, with any anticipated intraoperative difficulty prompting conversion. Consequently, the conversion rate in our series was 23%, which is within the expected range for centers in the early stages of adopting a robotic pancreatic surgery program [24].

However, according to the intention-to-treat principle, converted cases were analyzed within the robotic group. Thus, the outcomes reported for the robotic cohort also reflect those patients in whom the minimally invasive approach was ultimately not feasible. Importantly, no conversions were necessitated by emergent situations or intraoperative complications. This underscores the safety of our patient selection policy and supports the feasibility of implementing a robotic pancreaticoduodenectomy program, even during its initial phase. This relatively high conversion rate inevitably results in an increased economic burden. However, this should be viewed as part of the implementation costs inherent to the early phases of adopting a complex robotic procedure. This initial economic impact is expected and reflects a transitional phase during the learning curve, rather than the long-term cost profile of a mature and fully established robotic pancreaticoduodenectomy program.

Notably, the robotic group demonstrated a trend toward reduced estimated blood loss and shorter hospital stay, which, although not statistically significant, align with prior studies suggesting that the minimally invasive approach may attenuate surgical stress and accelerate recovery [6,7,26,31]. In our intention-to-treat analysis, the potential benefit of robotic-assisted surgery on the length of hospital stay might have been partially masked by patients converted to open surgery, whose postoperative course likely resembles that of primarily open procedures [32].

This study is based on data from a single center. While this design inherently limits the sample size, it also minimizes variability in surgical technique and postoperative management, reducing potential sources of bias. In this context, the homogeneity of care represents an additional strength of our single-center experience, enhancing the internal validity of the findings. Importantly, the study was conducted at a medium-volume center during the initial implementation phase of robotic surgery. Despite this, and the inclusion of an unselected patient population for age, or comorbidities, most of the perioperative outcomes fell within the international benchmark values established for pancreaticoduodenectomy in highly selected “benchmark” patients [33]. This finding is particularly noteworthy, as benchmark values are typically derived from expert centers operating under optimal conditions. The fact that our results align with these reference standards further supports the safety and effectiveness of robotic PD, even in real-world settings and during the early stages of institutional adoption.

The use of IPTW allowed us to create balanced pseudo-populations and reduce the effect of confounding variables—a crucial aspect when comparing surgical techniques outside of randomized controlled trials [25,28,31]. The balance achieved in baseline characteristics after IPTW strengthens the validity of these findings, particularly given the initial imbalances in Fistula Risk Score and ASA class [34].

This study has some limitations, with the first being its retrospective design. Also, despite IPTW adjustment, residual confounding cannot be excluded. Furthermore, the sample size is relatively small, particularly for detecting rare events such as grade C POPF or mortality. Nevertheless, the intention-to-treat inclusion of converted cases within the robotic group strengthens the validity of the findings. In addition, factors such as surgeon experience and evolving institutional preferences may have influenced the choice of surgical approach over time. While the inclusion criteria and selection policy for the robotic approach remained stable throughout the study period, a progressive increase in surgeon proficiency with the robotic technique may have contributed to broader indications in later years. This temporal dynamic, although not explicitly captured in the propensity score model, could have impacted outcomes and should be considered when interpreting the results.

## 5. Conclusions

In conclusion, robotic pancreaticoduodenectomy is a safe and effective technique in medium-volume centers. Despite longer operative times, it offers perioperative outcomes comparable to the open approach, with a trend toward shorter hospital stays. Future multicenter studies, ideally incorporating cost analyses and long-term oncological outcomes, are warranted to further define the role of robotic PD in routine clinical practice. Moreover, prospective registries may represent a feasible alternative to large-scale randomized trials, given the logistical challenges of such studies in this field.

## Figures and Tables

**Table 1:** Baseline characteristics of patients included in the study.

	Overall	Open	Robotic	*p*	SMD
Number of patients	74	31	43		
Age, years, median (IQR)	65.72 (58.19, 71.96)	66.00 (57.51, 72.96)	65.00 (60.47, 71.91)	0.653	0.124
Gender, female (%)	24 (32.4)	9 (29.0)	15 (34.9)	0.780	0.126
BMI, kg/m^2^, median (IQR)	23.18 (21.65, 25.89)	22.86 (21.84, 25.59)	23.44 (21.63, 26.05)	0.493	0.153
ASA category, *n* (%)				0.315	0.451
1	3 (4.1)	2 (6.5)	1 (2.3)		
2	31 (41.9)	15 (48.4)	16 (37.2)		
3	35 (47.3)	11 (35.5)	24 (55.8)		
4	5 (6.8)	3 (9.7)	2 (4.7)		
Preoperative biliary drainage, *n* (%)	38 (51.4)	13 (41.9)	25 (58.1)	0.254	0.328
Diagnosis, *n* (%)				0.074	0.969
Pancreatic ductal adenocarcinoma	42 (56.8)	17 (54.8)	25 (58.1)		
Extrahepatic cholangiocarcinoma	7 (9.5)	6 (19.4)	1 (2.3)		
Ampullary adenocarcinoma	11 (14.9)	2 (6.5)	9 (20.9)		
Duodenal cancer	2 (2.7)	1 (3.2)	1 (2.3)		
IPMN with worrisome features	4 (5.4)	1 (3.2)	3 (7.0)		
Neuroendocrine tumour	1 (1.4)	1 (3.2)	0 (0.0)		
Metastatic renal carcinoma	1 (1.4)	1 (3.2)	0 (0.0)		
Chronic pancreatitis	3 (4.1)	2 (6.5)	1 (2.3)		
Serous cystoadenoma	3 (4.1)	0 (0.0)	3 (7.0)		
Malignant tumour, *n* (%)	64 (86.5)	28 (90.3)	36 (83.7)	0.635	0.197
Tumour primary site: pancreas, *n* (%)	42 (56.8)	17 (54.8)	25 (58.1)	0.964	0.067
Preoperative chemotherapy, *n* (%)	13 (17.6)	7 (22.6)	6 (14.0)	0.514	0.225
Vascular resection, *n* (%)	7 (10.9)	3 (13.6)	4 (9.5)	0.937	0.129
Fistula risk score, median (IQR)	4 (2, 6)	4 (2, 6)	3 (2, 4.5)	0.265	0.250

IQR, Interquartile range; SMD, Standardized Mean Difference; ASA, American Society of Anesthesiologists; BMI, Body Mass Index.

**Table 2:** Comparison of baseline characteristics of patients undergoing open and roboticpancreaticoduodenectomy before and after IPTW.

	Before IPTW				After IPTW			
	Open	Robotic	*p*	SMD	Open	Robotic	*p*	SMD
Number of patients	31	43			31.27	42.75		
Age, years (median [IQR])	66.00 (57.51, 72.96)	65.00 (60.47, 71.91)	0.653	0.124	65.31 (56.76, 69.97)	64.63 (60.93, 71.89)	0.987	0.006
Gender, female (%)	9 (29.0)	15 (34.9)	0.780	0.126	10.6 (34.0)	16.9 (39.4)	0.678	0.112
BMI, kg/m^2^ (median [IQR])	22.86 (21.8, 25.59)	23.44 (21.63, 26.05)	0.493	0.153	22.86 (21.52, 25.61)	23.22 (21.63, 25.87)	0.758	0.049
ASA category, *n* (%)			0.315	0.451			0.998	0.044
1	2 (6.5)	1 (2.3)			1.2 (3.8)	1.3 (3.1)		
2	15 (48.4)	16 (37.2)			12.8 (40.9)	17.8 (41.6)		
3	11 (35.5)	24 (55.8)			15.1 (48.3)	20.5 (47.9)		
4	3 (9.7)	2 (4.7)			2.2 (6.9)	3.2 (7.5)		
Preoperative biliary drainage, *n* (%)	13 (41.9)	25 (58.1)	0.254	0.328	12.0 (38.5)	25.7 (60.1)	0.089	0.442
Diagnosis, *n* (%)			0.074	0.969			0.054	1.003
Pancreatic ductal adenocarcinoma	17 (54.8)	25 (58.1)			18.6 (59.6)	25.1 (58.8)		
Extrahepatic cholangiocarcinoma	6 (19.4)	1 (2.3)			5.3 (17.0)	0.9 (2.0)		
Ampullary adenocarcinoma	2 (6.5)	9 (20.9)			1.1 (3.6)	9.9 (23.1)		
Duodenal cancer	1 (3.2)	1 (2.3)			0.6 (2.1)	1.4 (3.2)		
IPMN	1 (3.2)	3 (7.0)			1.9 (6.1)	2.5 (5.8)		
Neuroendocrine tumour	1 (3.2)	0 (0.0)			1.3 (4.2)	0.0 (0.0)		
Metastatic renal carcinoma	1 (3.2)	0 (0.0)			0.7 (2.2)	0.0 (0.0)		
Chronic pancreatitis	2 (6.5)	1 (2.3)			1.7 (5.4)	0.7 (1.7)		
Serous cystoadenoma	0 (0.0)	3 (7.0)			0.0 (0.0)	2.3 (5.5)		
Malignant tumour, *n* (%)	28 (90.3)	36 (83.7)	0.635	0.197	27.7 (88.5)	37.2 (87.0)	0.861	0.045
Tumour site: pancreas, *n* (%)	17 (54.8)	25 (58.1)	0.964	0.067	18.6 (59.6)	25.1 (58.8)	0.948	0.016
Preoperative chemotherapy, *n* (%)	7 (22.6)	6 (14.0)	0.514	0.225	5.6 (18.0)	8.2 (19.2)	0.908	0.029
Vascular resection, *n* (%)	3 (13.6)	4 (9.5)	0.937	0.129	2.6 (12.3)	3.4 (8.3)	0.597	0.133

IPTW, Inverse Probability Treatment Weighting; IQR, Interquartile range; SMD, Standardized Mean Difference; ASA, American Society of Anes thesiologists; BMI, Body Mass Index.

**Table 3:** Comparison of outcomes of patients undergoing open and robotic pancreaticoduodenectomy before and after IPTW.

	Before IPTW			After IPTW		
	Open	Robotic	*p*	Open	Robotic	*p*
Number of patients	31	43		31.27	42.75	
Operative time, min, median (IQR)	480.00 (385.00, 525.00)	540.00 (480.00, 600.00)	0.004	479.40 (393.18, 498.00)	540.00 (471.10, 600.00)	0.009
Estimated blood loss, ml, median (IQR)	250.00 (125.00, 375.00)	200.00 (150.00, 500.00)	0.679	250.00 (100.00, 303.52)	200.00 (150.00, 450.00)	0.631
Delayed gastric emptying (grade B/C), *n* (%)	4 (12.9)	9 (20.9)	0.558	4.1 (13.0)	8.5 (19.9)	0.464
Clinically relevant POPF (grade B/C), *n* (%)	8 (25.8)	8 (18.6)	0.648	7.1 (22.7)	8.0 (18.6)	0.675
Biliary leak, *n* (%)	1 (3.2)	4 (9.3)	0.577	1.0 (3.1)	3.7 (8.6)	0.334
Post-pancreatectomy hemorrhage (grade C), *n* (%)	3 (9.7)	5 (11.6)	1.000	2.5 (8.0)	4.1 (9.6)	0.797
Reoperation, *n* (%)	5 (16.1)	8 (18.6)	1.000	4.3 (13.8)	7.3 (17.0)	0.708
Patients with complications Grade ≥ IIIa, *n* (%)	10 (32.3)	14 (32.6)	1.000	9.0 (28.9)	13.1 (30.6)	0.880
90 days mortality (%)	1 (3.2)	2 (4.7)	1.000	1.5 (4.9)	2.1 (4.9)	0.998
Length of hospital stay, days, median (IQR)	20.00 (14.00, 29.00)	20.00 (12.50, 32.00)	0.960	20.00 (14.00, 28.66)	17.00 (11.96, 30.47)	0.686
Readmission, *n* (%)	7 (24.1)	4 (10.0)	0.211	7.2 (25.1)	3.7 (9.4)	0.095
Lymph nodes retrieved, median (IQR)	20.00 (14.50, 27.50)	20.00 (13.00, 27.50)	0.709	20.00 (14.37, 28.30)	20.00 (13.06, 27.05)	0.579
R status, *n* (%)			0.550			0.588
R0	26 (83.9)	34 (79.1)		25.1 (80.2)	35.5 (83)	
R1	2 (6.5)	1 (2.3)		2.6 (8.4)	1 (2.3)	
R2	0	1 (2.3)		0	0.8 (1.8)	
NA	3 (9.7)	7 (16.3)		3.6 (11.5)	5.5 (13)	

IPTW, Inverse Probability Treatment Weighting; IQR, Interquartile range; POPF, postoperative pancreatic fistula.

## Data Availability

The data presented in this study are available on request from the corresponding author due to legal reasons.

## Abbreviations

The following abbreviations are used in this manuscript:

ASA	American Society of Anesthesiologists
BMI	Body Mass Index
DGE	Delayed Gastric Emptying
IPTW	Inverse Probability of Treatment Weighting
ISGPS	International Study Group of Pancreatic Surgery
LOS	Length of Stay
PD	Pancreaticoduodenectomy
POPF	Postoperative Pancreatic Fistula
PPH	Post-Pancreatectomy Hemorrhage
SMD	Standardized Mean Difference
